# Corrigendum: Multi-Omics Analysis in Initiation and Progression of Meningiomas: From Pathogenesis to Diagnosis

**DOI:** 10.3389/fonc.2020.630455

**Published:** 2020-12-14

**Authors:** Jiachen Liu, Congcong Xia, Gaiqing Wang

**Affiliations:** ^1^ Clinical Medicine, Xiangya Medical College of Central South University, Changsha, China; ^2^ Department of Neurology, Sanya Central Hospital (The Third People’s Hospital of Hainan Province), Sanya, China

**Keywords:** meningiomas, biomarker, genomics, epigenomics, transcriptomics, proteomics

In the published article, affiliation 3 “Department of Neurology, Shanxi Medical University, Taiyuan, China” was added in error. It should be deleted.

The authors apologize for this error and state that this does not change the scientific conclusions of the article in any way. The original article has been updated.

